# High seroprevalence of *Strongyloides stercoralis* among individuals from endemic areas considered for solid organ transplant donation: A retrospective serum-bank based study

**DOI:** 10.1371/journal.pntd.0007010

**Published:** 2018-11-29

**Authors:** Joan Gómez-Junyent, David Paredes, Juan Carlos Hurtado, Ana Requena-Méndez, Angel Ruiz, Maria Eugenia Valls, Jordi Vila, Jose Muñoz

**Affiliations:** 1 ISGlobal, Barcelona Ctr. Int. Health Res. (CRESIB), Hospital Clínic—Universitat de Barcelona, Barcelona, Spain; 2 Donation and Transplant Coordination Section, Hospital Clínic–Universitat de Barcelona, Barcelona, Spain; 3 Department of Clinical Microbiology, Centre for Biomedical Diagnosis, Hospital Clínic, Barcelona, Spain; George Washington University School of Medicine and Health Sciences, UNITED STATES

## Abstract

**Background:**

*Strongyloides stercoralis* is a worldwide disseminated parasitic disease that can be transmitted from solid organ transplant (SOT) donors to recipients. We determined the serological prevalence of *S*. *stercoralis* among deceased individuals from endemic areas considered for SOT donation, using our institution’s serum bank.

**Methodology:**

Retrospective study including all deceased potential donors from endemic areas of strongyloidiasis considered for SOT between January 2004 and December 2014 in a tertiary care hospital. The commercial serological test IVD-Elisa was used to determine the serological prevalence of *S*. *stercoralis*.

**Principal findings:**

Among 1025 deceased individuals during the study period, 90 were from endemic areas of strongyloidiasis. There were available serum samples for 65 patients and 6 of them tested positive for *S*. *stercoralis* (9.23%). Only one of the deceased candidates was finally a donor, without transmitting the infection.

**Conclusions:**

Among deceased individuals from endemic areas considered for SOT donation, seroprevalence of strongyloidiasis was high. This highlights the importance of adhering to current recommendations on screening for *S*. *stercoralis* among potential SOT donors at high risk of the infection, together with the need of developing a rapid diagnostic test to fully implement these screening strategies.

## Author summary

Strongyloidiasis is a neglected tropical disease caused by a parasite which is endemic in most parts of the world. It can cause a life-threatening disease among immunosuppressed individuals and can be transmitted from solid organ transplant donors to recipients. We retrospectively investigated the prevalence of strongyloidiasis among deceased individuals from endemic areas who were considered for solid organ transplant donation in our center, by performing a serological assay using our institution’s serum bank. We found a high prevalence of strongyloidiasis among these deceased candidates to donation, but only one of the six who tested positive was finally a donor, without transmitting the disease to recipients. Our results should encourage physicians to adhere to current guidelines which recommend active screening of strongyloidiasis in potential solid organ transplant donors from endemic areas who may be infected. There is a clear need for a rapid diagnostic test to fully implement systematic screening among these individuals.

## Introduction

*Strongyloides stercoralis* is a parasitic infection which is endemic in large parts of Latin America, Asia and Africa with an estimated prevalence of more than 400 million infected [1]. Due to its characteristic life cycle, individuals can carry the infection lifelong with mild or no symptoms, unless treated. However, strongyloidiasis can result in a life-threatening disease in immunosuppressed individuals, such as solid organ transplant (SOT) recipients under immunosuppressive drugs and/or steroids [2].

Most commonly, individuals become infected by contacting infective larvae when walking barefoot. In recent years, cases of transmission between SOT donors to recipients have been reported [3–7]. In these reports, strongyloidiasis in donors was commonly missed by physicians, possibly due to asymptomatic carriage and/or failure to diagnose severe forms of the disease. Prior testing or screening in donors had not been performed.

Currently, the American Society of Transplantation recommends systematic screening of *S*. *stercoralis* in donors and candidate recipients from endemic areas and/or with unexplained eosinophilia [8]. However, the increasing reports of transmission from donors to recipients suggest that these guidelines may not be followed by all Organ Procurement Organizations (OPO) worldwide. Data from the New York Organ Donor Network showed a strongyloidiasis serological prevalence of 4.3% among 233 eligible potential donors screened [9]. However, there are no other data published on strongyloidiasis prevalence among deceased donors considered for SOT.

Thus, the aim of this study was to determine the serological prevalence of strongyloidiasis among candidates to SOT donation from endemic areas in a tertiary care hospital in Spain with authorization for organ donor procurement and transplantation.

## Methods

This is a retrospective study including all deceased individuals from endemic areas for strongyloidiasis who were considered for organ donation between January 2004 and December 2014 in Hospital Clínic, a tertiary care hospital in Barcelona, Spain. Although the prevalence rates are probably very different within continents and countries, including rural and urban areas, donors born in Latin America, Africa and Asia were considered to be from an endemic area for strongyloidiasis. The main objective of the study was to determine the seroprevalence of *S*. *stercoralis* among candidates for deceased organ donation. This study was approved by the hospital’s Ethics Committee.

Since 2004, the Transplantation Coordination Unit has been registering in a database all deceased individuals who are considered for deceased organ donation. A questionnaire is filled with demographic data (age, sex and country of origin), comorbidities (arterial hypertension, diabetes mellitus, HIV and HCV infection) and organ procurement data (cause of death, absolute or relative contraindications for organ donation and organs procured). Deceased organs are only procured if the donor does not have absolute contraindication for donation like active disseminated cancer or disseminated infection from any origin, with multiple organ failure, and after family consent from the deceased donor. Several microbiological serologies are performed to exclude potentially transmittable diseases and a frozen serum sample from all deceased organ donors remain under custody in the Microbiology Department of the hospital, for at least 30 years after donation.

Using the serum samples of potential donors from endemic areas for strongyloidiasis, we performed a *S*. *stercoralis* serological test. The commercial test IVD-ELISA (IVD Research, Carlsbad, CA), which detects IgG antibodies by using somatic antigens from larvae of the parasite, was used (sensitivity 91.2%, specificity 99.1% [10]). Currently, IVD-ELISA is the available test in our hospital. The sample is defined as positive if the absorbance/0.2 (*i*) > 1.1. All analyzed samples were anonymized.

Stata version 13.1 (Stata Corporation, College Station, TX, USA) was used for statistical analyses. Categorical variables were described by counts and percentages, whereas continuous variables were expressed as means and standard deviations (SD) or medians and interquartile ranges (IQRs).

## Results

During the period 2004–2014, 1025 deceased individuals were evaluated as potential deceased organ donors. Of these, 90 donors (8.78%) were native from endemic areas for strongyloidiasis. Most were males (63/90 cases; 70%) and median age of all cases was 41 years old (IQR 33–51). More than half of the deceased organ donors from endemic areas had been born in Latin America (49/90 cases; 54.44%), whereas the rest were from Southeast Asia (24/90 cases; 26.67%) and Africa (17/90 cases; 18.89%).

Finally, only in 65 of the 90 cases, available serum samples were found and analysed for *S*. *stercoralis* serology and 6/65 cases (9.23%) were found to have a positive test for strongyloidiasis. *S*. *stercoralis* serology indices ranged between 1.84 and 9.32. As shown in Table 1, four were male and age range was between 22 and 62 years. From the 6 positive cases, three individuals were native from Latin America (2 from Brazil and 1 from Ecuador), two from Africa (Senegal and Ghana) and one from Southeast Asia (Philippines). Between the seropositive deceased donors, only one was considered suitable for donation and 2 kidneys, two lungs, heart and liver were procured and transplanted.

**Table 1:** Main characteristics, final donation status and serology index among six deceased individuals considered for solid organ donation who tested positive for strongyloidiasis.

Candidate	Year	Age/ Sex	Country of origin	Cause of death	Final donation status	Serology index
1	2007	62 / F	Brazil	Head trauma	Yes (kidney, liver, lungs, heart)	2.26
2	2008	22 / M	Ghana	Subarachnoid haemorrhage	No (medical contraindication)	2.36
3	2011	36 / M	Senegal	Cardiovascular	No (medical contraindication)	1.84
4	2012	62 / F	Philippines	Cardiovascular	No (organ problems)	9.32
5	2012	38 / M	Brazil	Cardiovascular	No (organ problems)	9.13
6	2012	42 / M	Ecuador	Head trauma	No (judicial refusal)	2.42

F: Female; M: Male.

Given these results, we investigated whether there had been transmission of strongyloidiasis to SOT recipients from the infected deceased donor. Transplant units from other centres were informed and we were only able to evaluate two recipients who were transplanted in our hospital in 2007. They were a 50-year-old and 55-year-old male recipients who had received a kidney and liver transplant, respectively. Strongyloidiasis serology and stool culture were negative in both recipients.

## Discussion

In this article, we found that the seroprevalence of strongyloidiasis among deceased individuals from endemic areas who are evaluated as potential donors was high. Apart from the data published by Abanyie et al. [9], there are no previous reports of the seroprevalence of strongyloidiasis from OPOs’ or Procurement Hospital serum banks.

Strongyloidiasis can be transmitted from donors to SOT recipients, although this is probably uncommon. The true risk of transmission and related factors are difficult to determine, although the parasite burden and the stage of the infection could play an important role [7]. However, since most SOT recipients will be immunosuppressed for the rest of their lives, the possibility of becoming infected by transmission from the donor represents a serious public health issue.

AST guidelines clearly recommend screening both donors and recipients from endemic areas [8], but this is probably not universally followed in most procurement hospitals and centers with SOT programs in USA or Europe, as has been recently published [11]. Reasons for this may be related with little number of donors and/or recipients native from endemic areas, the lack of standardized serological tests in some laboratories or economic issues. Despite all these potential difficulties, physicians in charge of procurement centers and SOT programs should definitely incorporate AST recommendations when testing for donor-to-recipient transmissible diseases.

In our hospital, following the present study results and a previous reported case of transmission [7], screening policies have been redefined. Clinicians in charge of transplantation programs have been encouraged either to screen potential recipients at risk or refer them to the Tropical Medicine Department Outpatient Clinic. A similar approach has been suggested for donors who may undergo elective transplant surgery. Despite clinicians’ awareness of the problem, more difficulties have been faced in implementing an efficient strongyloidiasis screening strategy in urgent donations, given the characteristics of these procedures and the current available diagnostic tests. In every case, a strict protocol is currently followed according to the individualized donor’s or recipient’s risk of infection, prompting exhaustive investigations prior or during the SOT donation, with rapid antiparasitic treatment initiation, if necessary.

Strongyloidiasis, a neglected tropical disease, is one of the most prevalent infections worldwide. In recent years, it has become an important health problem in non-endemic settings, where high prevalence in specific populations has been encountered [12, 13]. The increasing frequency of migration flows of people from endemic areas in recent years may have resulted in these individuals becoming SOT donors more often. Similarly, many endemic countries have national transplant programs and the number of organ procurements may increase in the coming years. Clinicians should also take into account that strongyloidiasis has been described in temperate areas, such as Spain, especially among the elderly [14], who may be potential recipients prone to develop severe forms if immunosuppressed. All these considerations make it even more reasonable to incorporate AST recommendations in the daily clinical practice of deceased and living donors and SOT recipients in endemic and non-endemic settings.

In this line, the available serological tests currently available are far from being optimal [10]. Two commercial tests (IVD-ELISA and Bordier-ELISA) based on Enzyme-Linked Immunosorbent Assay (ELISA) are among the most common used tests. These present high sensitivity (73–100%), which can be lower in immunocompromised individuals [15]. Moreover, these tests show cross-reactivity with other nematode infections, potentially causing false-positive results. In our study, cross-reactivity was probably limited, since positive samples had high serological indices [10]. Another limitation of these tests is that they are often time-consuming and require specific expertise, which may add difficulties to systematic screening in the urgency of transplantation.

All these inconvenients in the serological diagnosis of strongyloidiasis support the importance of focusing research efforts on the development of better diagnostic tools in the near future [16]. It is clear that there is a need for a rapid, validated and robust test, if systematic screening needs to be implemented among the deceased and living donors and SOT recipient population.

Our study has several limitations. It was a retrospective study performed in a single centre, thereby limiting the generalizability of the results. Apart from the high number of missing serum samples, the lower proportion of individuals from Africa and Asia may have affected our results. Finally, the existence of false positives due to a co-infection by other nematodes is also difficult to rule out.

In conclusion, we found a high seroprevalence of strongyloidiasis in individuals from endemic settings evaluated as potential deceased donors for SOT. Our data reinforce the importance of following current guidelines recommending systematic screening of potential donors from endemic areas. More research is urgently needed to develop rapid diagnostic tests which can be used in daily clinical practice.

## Supporting information

S1 Checklist STROBE checklist. (PDF)
